# Biotechnological Potential of Different Organs of Mistletoe (*Viscum album* L.) Collected from Various Host Tree Species in an Urban Area

**DOI:** 10.3390/plants11202686

**Published:** 2022-10-12

**Authors:** Liubov Skrypnik, Pavel Feduraev, Anton Golovin, Pavel Maslennikov, Nikolay Belov, Matvei Matveev, Artem Pungin

**Affiliations:** MedBio Cluster, Immanuel Kant Baltic Federal University, 236040 Kaliningrad, Russia

**Keywords:** medicinal plants, secondary metabolites, biological active compounds, raw material, hemiparasitic plants, plant–plant interactions

## Abstract

From an economic and ecological standpoint, it is crucial to investigate the biologically active compounds of mistletoe plants, which are currently discarded by pruning urban mistletoe-infested trees. In the present study, the content of phenolic compounds, triterpenic and organic acids, as well as the antioxidant activity of the extracts of various mistletoe organs (leaves, stems, and fruits) collected from the most infested tree species were investigated. The mistletoe samples collected from *Betula pendula, Acer platanoides, Crataegus monogyna*, and *Sorbus aucuparia* showed the highest content of phenolic acids and flavonoids as well as antioxidant activity, as measured by 2,2-diphenyl-1-picrylhydrazyl (DPPH), 2,2′-azino-bis(3-ethylbenzothiazoline-6-sulfonic acid (ABTS), and ferric reducing/antioxidant power (FRAP) assays. The leaves and stems of mistletoe from *Tilia cordata* were characterized by a high content of triterpenic acids (oleanolic, ursolic, and betulinic). The leaves and fruits of mistletoe plants from *Populus nigra* and *Salix alba* contained a high concentration of organic acids, particularly succinic and citric acids. Compared to stem and leaf extracts, the antioxidant activity of the mistletoe fruit extracts was 1.5–3 times higher. The obtained results indicate that mistletoe is a valuable raw material and can be used as a source of phenolic compounds and triterpenic and organic acids, as well as for producing extracts with antioxidant properties.

## 1. Introduction

Mistletoe (*Viscum album* L.) is a hemiparasitic plant that develops stable haustoria in the host tree. According to studies conducted over the past few years, the intensity of mistletoe damage on trees in European countries increases from year to year [1,2,3,4,5]. Mistletoe infection causes damage to forests, gardens, plantations, and ornamental trees. The affected host trees suffer from decreased growth and vigour, reduced fruiting, quality and quantity of wood, as well as weakened resistance to insect and fungal infestation [1,6]. Despite the fact that biological methods have recently been studied to slow down the spread of mistletoe, the only method of reducing the amount of mistletoe in urban and agricultural areas remains the mechanical removal of affected branches or the complete removal of trees [7,8]. Often, however, mistletoe bushes, made from these branches and trees, serve no purpose and are considered low-value waste. The study of the potential application of this waste in the production of valuable biological products or the isolation of biologically active components is important from an economic and ecological point of view.

Various parts of mistletoe have been widely used in folk medicine for a long time. Mistletoe is used for the prevention and treatment of various diseases, such as atherosclerosis, hypertension, arthritis, bronchial asthma, inflammatory kidney diseases, diabetes mellitus, etc. Potentially, mistletoe extracts can also be used as hepatoprotective or sedative drugs [9,10,11,12]. A significant number of studies are related to the study of the effectiveness of mistletoe and its phytocomponents for application in the treatment of cancer [13,14,15,16].

The most well-studied and most active phytocomponents of mistletoe include lectins and viscotoxins, which play an essential role in the treatment of cancer due to their apoptotic and cytotoxic effects. It has been shown that both groups also have an immunomodulatory effect [17,18]. Another group of compounds identified in mistletoe includes phenolic acids, phenylpropanoids, and flavonoids, which have antioxidant and anti-inflammatory activity and can lower blood pressure [19,20,21]. In addition, triterpenic acids, in particular, oleanolic, ursolic, and betulinic acids, which have cytotoxic and apoptotic properties, have been identified in mistletoe [22,23,24]. Phytochemical studies of mistletoe have also revealed the presence of other important pharmacological compounds, such as phytosterols, alkaloids, oligopeptides, polysaccharides, and fatty acids [25,26].

The accumulation of secondary metabolites in plants is influenced by many factors related to both the location and growing conditions as well as the growth and development of the plant itself [27]. It was previously shown that the metabolic profile of mistletoe also depends on the tree on which it grows [28,29,30,31]. In addition, the qualitative and quantitative composition of phytocomponents in different plant organs can vary greatly, which, in turn, causes a difference in their biological activity [18,32].

Previous studies have shown that mistletoe prevalence is especially high in an urban environment, characterized by a number of factors unfavourable for tree growth [33]. In studies conducted in 2019–2021 on the territory of Kaliningrad, it was shown that the species most affected by mistletoe were *Tilia cordata* Mill., *Acer platanoides* L., *Populus nigra* L., *Acer saccharinum* L., *Salix alba* L., *Crataegus monogyna* Jacq., *Sorbus aucuparia* L., and *Betula pendula* Roth. On average, there were more than 10 mistletoe bushes per tree [3]. In this regard, the purpose of this study was to evaluate the content of some groups and individual phenolic compounds, triterpenic and organic acids, as well as the antioxidant activity of extracts of various mistletoe organs (leaves, stems, and fruits) collected from the most-infested tree species. The obtained results will make it possible to assess the potential of mistletoe, including current waste from pruning urban trees, as a source of valuable biologically active compounds.

## 2. Results

### 2.1. Variation in the Content of Phenolic Compounds

The results of the two-way ANOVA of the total content of phenolic compounds, flavonoids, and hydroxycinnamic acids in the mistletoe samples collected from various species of host trees showed that the level of these compounds was significantly influenced by both the type of tree and the analysed organ of mistletoe (Table 1).

The samples collected from *C. monogyna*, *A. platanoides*, *S. aucuparia*, *S. alba*, and *B. pendula* were distinguished by a high total content of phenolic compounds. The average content of phenolic compounds in these mistletoe samples collected from these tree species ranged from 12.34 to 13.44 mg GAE g^–1^ DW. The lowest total content of phenolic compounds was determined in mistletoe samples collected from *A. saccharinum* (9.74 mg GAE g^–1^ DW). The study of phenolic compound accumulation in various organs of mistletoe showed that the fruits contained 3.0–3.2 times more phenolic compounds compared to leaves and stems (Table 1).

Mistletoe samples collected from *B. pendula*, *C. monogyna*, *S. aucuparia*, and *A. platanoides* were characterized by a higher content of flavonoids (0.97–1.26 mg QE g^–1^ DW) compared to samples collected from other host tree species. Among the studied mistletoe organs, the maximum content of flavonoids was found in leaves (1.21 mg QE g^–1^ DW). The content of flavonoids in stems and fruits was 1.6 times lower (Table 1).

Mistletoe samples collected from *B. pendula* were also characterized by the maximum total content of hydroxycinnamic acids, which amounted to 2.48 mg CAE g^–1^ DW. A study of the accumulation of hydroxycinnamic acids in various organs of mistletoe showed that the maximum content of hydroxycinnamic acids was in leaves and stems, and the lowest in fruits (Table 1).

In general, mistletoe samples collected from *A. saccharinum* and *P. nigra* were characterized by a lower total content of phenolic compounds, flavonoids, and hydroxycinnamic acids.

**Figure A1:**
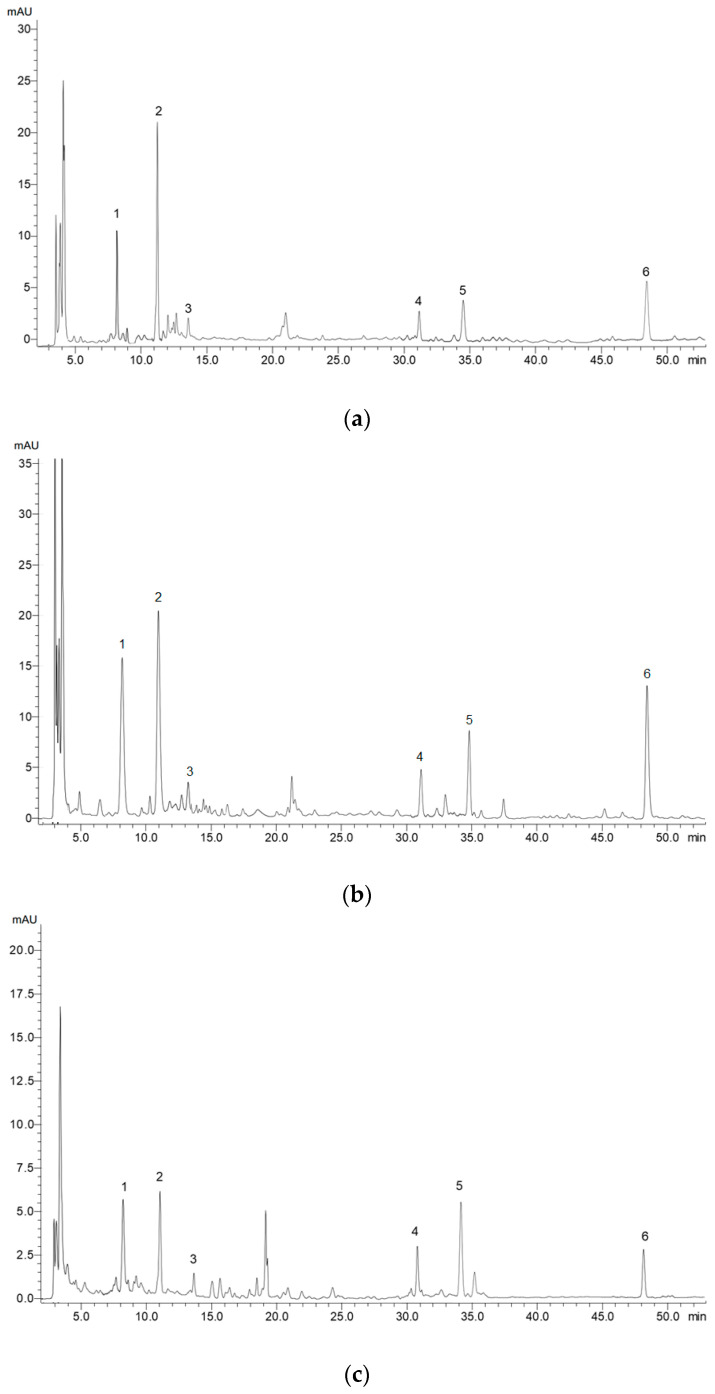
Exemplary HPLC chromatogram of the phenolic acids and flavonoids in (**a**) stems, (**b**) leaves, and (**c**) fruits of mistletoe (*Viscum album* L) harvested from *B. pendula*. 1—neochlorogenic acid; 2—chlorogenic acid; 3—caffeic acid; 4—isorhamnetin; 5—kaempferol derivative; 6—quercetin derivative.

Based on the results of the high-performance liquid chromatography (HPLC) analysis, it was found that although the content of phenolic acids and flavonoids were significantly dependent on the host tree and the mistletoe organ, the phenolic compound composition was similar in all samples. Among the phenolic compounds in the samples, chlorogenic and neochlorogenic acids, as well as flavonol derivatives (isorhamnetin and derivatives of quercetin and kaempferol) dominated. Examples of the chromatograms are shown in Figure A1 (Appendix A).

Mistletoe samples collected from *B. pendula*, *A. platanoides*, and *C. monogyna* were distinguished by a high content of chlorogenic and neochlorogenic acids. The maximum contents of isorhamnetin and kaempferol derivatives were found in samples of mistletoe from *B. pendula*. A high content of the quercetin derivative was detected in mistletoe samples collected from *T. cordata*, *A. platanoides*, *B. pendula*, *C. monogyna*, *S. alba*, and *S. aucuparia* (Table 2).

Leaves and stems of mistletoe were characterized by about a 1.5–2 times higher content of neochlorogenic acid, chlorogenic acid, and quercetin derivative compared to fruits. For the rest of the studied phenolic compounds, the differences between organs were insignificant (Table 2).

### 2.2. Variation in the Content of Triterpenic Acids

The separation and quantitative determination of triterpenic acids (ursolic, oleanolic, and betulinic acids) were carried out by high-performance thin layer chromatography (HPTLC). The quantitative content of triterpenic acids significantly depended on both the type of host tree and mistletoe organ (Table 3). Oleanolic acid dominated among the triterpenic acids in mistletoe, the content of which was about 3–5 times higher compared to ursolic acid and 6–8 times higher compared to betulinic acid. The highest content of ursolic acid was found in mistletoe samples collected from *S. alba* and *T. cordata*; oleanolic acid—in mistletoe samples collected from *T. cordata*; and betulinic acid—in mistletoe samples collected from *P. nigra*, *S. alba*, and *T. cordata.* Leaves and stems of mistletoe were characterized by a significantly higher content of triterpenic acids compared to fruits (Table 3).

### 2.3. Variation in the Content of Organic Acids

The separation and quantitative determination of organic acids, in particular succinic, citric, oxalic, formic, fumaric, propionic, malic, and sorbic acids, were carried out in mistletoe samples by the capillary electrophoresis method (Table 4).

Based on the results of the two-way ANOVA, it was found that the content of organic acids in mistletoe was influenced by the type of host tree and mistletoe organ (Table 4). Mistletoe samples collected from *P. nigra* were characterized by a significantly higher content of succinic, citric, fumaric, and malic acids. High content of succinic, oxalic, and formic acids was found in mistletoe collected from *S. alba*. The samples collected from *C. monogyna*, in addition to their high content of oxalic and malic acids, were characterized by the highest content of propionic acid (Table 4).

In general, both leaves and fruits of mistletoe were characterized by a high content of organic acids. In particular, significantly higher contents of succinic, citric, and propionic acids were observed in these organs in comparison with stems. Mistletoe leaves were also distinguished by a higher content of formic, fumaric, and malic acids compared with stems. No malic acid was found in the fruits of any of the mistletoe samples. Sorbic acid, on the contrary, was contained mainly in the fruits and stems of mistletoe.

### 2.4. Antioxidant Activity of Mistletoe Extracts

The antioxidant activity of mistletoe extracts significantly depended on the type of host tree and on the mistletoe organ (Table 5). In general, higher antioxidant activity was observed in mistletoe samples collected from *S. aucuparia*, *C. monogyna* (according to the DPPH and FRAP assays), *T. cordata* and *A. platanoides* (according to the DPPH assay), and *B. pendula* (according to the ABTS and FRAP assays). By determining antioxidant activity using these three methods, it was shown that the antioxidant activity of mistletoe fruit extracts was 1.5–3 times higher compared to the antioxidant activity of stems and leaf extracts (Table 5).

### 2.5. Heat Map and Cluster Analysis of the Mistletoe Samples and Phytochemical Parameters

**Figure 1:**
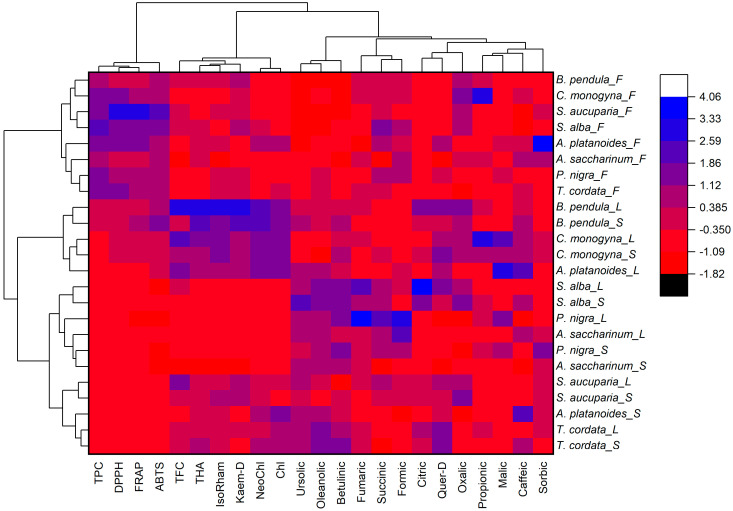
Heat map with clusters of the studied phytochemical parameters (at the top) and mistletoe samples (at the left). TPC—total phenolics content; TFC—total flavonoids content; THA—total hydroxycinnamic acids; IsoRham—isorhamnetin; Quer-D—quercetin derivative; Kaem-D—kaempferol derivative; DPPH—antioxidant activity determined by DPPH (2,2-diphenyl-1-picrylhydrazyl) assay; ABTS—antioxidant activity determined by ABTS (2,2′-azino-bis(3-ethylbenzothiazoline-6-sulfonic acid)) assay; FRAP—ferric reducing/antioxidant power. The uppercase letters following the host tree species indicate F—fruits, L—leaves, and S—stems.

Based on the normalized values of the studied parameters, a heat map with cluster analysis was built (Figure 1). The dendrogram presented in Figure 1 (top) demonstrates that all the studied parameters can be divided into three main clusters. The first cluster included the total content of phenolic compounds and antioxidant activity (DPPH, ABTS, and FRAP assays). The second cluster included the total content of flavonoids, hydroxycinnamic acids, the content of isorhamnetin, kaempferol derivative, chlorogenic, and neochlorogenic acids. The third cluster included triterpenic acids, organic acids, caffeic acid, and a quercetin derivative.

The dendrogram given on the left shows that the studied mistletoe samples can be divided into two main clusters (Figure 1, left). The first cluster included all samples of mistletoe fruits collected from various species of host trees. The second included leaves and stems of mistletoe. It is worth noting that cluster analysis did not allow mistletoe samples collected from a certain species of tree to be isolated into a separate cluster.

## 3. Discussion

### 3.1. Impact of Host Tree Species on the Content of Phytochemicals in Mistletoe

Mistletoe belongs to semi-parasitic plants, and therefore depends on the transfer of nutrients from the host tree. Mistletoe is believed to absorb water, mineral nutrients, and carbohydrates (glucose, fructose, and sucrose) from host trees [34]. As a result, the qualitative and quantitative composition of phytocomponents in mistletoe, and its biological activity, strongly depend on the type of tree on which the mistletoe grows. The results obtained in this study confirm this hypothesis. According to the results of the ANOVA, the host tree species had a significant effect on the accumulation of all the studied compounds and antioxidant activity (Table 1, Table 2, Table 3, Table 4 and Table 5).

Among the eight studied host trees, the samples of mistletoe growing on *B. pendula*, *A. platanoides*, *C. monogyna*, and *S. aucuparia* were distinguished by a higher content of both some groups of phenolic compounds and individual phenolic acids and flavonoids. Mistletoe samples collected from *A. saccharinum* and *P. nigra*, on the contrary, were characterized by a lower content of phenolic compounds. The results obtained in this study are partially consistent with the results presented in [31]. In particular, Pietrzak and Nowak (2021) also found a high total content of phenolic compounds in mistletoe collected from *C. monogyna* and *S. aucuparia.* However, mistletoe with *A. platanoides* was characterized by a low content of phenolic compounds, whereas in mistletoe with *P. nigra*, on the contrary, their content was high [31]. The differences in the obtained data are probably due to the fact that different mistletoe organs were used for the analysis and the studied plants grow in different ecological and climatic conditions. It is well established that the synthesis and proper accumulation of secondary metabolites, including phenolic compounds, are strictly controlled in a spatial and temporal manner and influenced by the changing abiotic and biotic environment [27].

The highest content of oleanolic acid, the dominant acid among the studied triterpenic acids, was found in mistletoe samples collected from *T. cordata.* Similar results were obtained in the work by Wójciak-Kosior et al. (2017) [35]. It is worth noting, however, that the average oleanolic acid content measured in mistletoe samples from *T. cordata* in the present work was 1.4 times lower compared to data available in previous studies (6.18 mg g^–1^ and 8.62 mg g^–1^, respectively) [35]. The amount of pentacyclic triterpenoids in plants is not constant and can significantly vary, depending on the activity of the enzyme systems and external factors. Changes in pentacyclic triterpenoid concentrations in plant sources may be related to a specific climate, season, landscape, and cultivation strategies [36].

Organic acids are widely distributed in fruits, vegetables, and herbs. Previous studies have shown that organic acids can have an antioxidant effect by directly removing free radicals and by complexing metal ions that contribute to the formation of free radicals [37]. In addition, organic acids are important antimicrobial compounds [38]. The information in previous studies on the qualitative and quantitative composition of organic acids in mistletoe is insufficient. In this study, succinic, citric, oxalic, formic, fumaric, propionic, malic, and sorbic acids were separated and quantified using capillary electrophoresis. It is shown that succinic and citric acids on average predominated in mistletoe samples (5.32 and 2.12 mg g^–1^, respectively) (Table 4). In addition, it was revealed for the first time that the content of the studied organic acids was significantly influenced by the host tree species. According to some studies, mistletoe can absorb not only water, mineral salts, and carbohydrates from the host tree, but also organic acids [39]. This fact can explain the significant influence of the host tree species on their composition and content in the mistletoe.

Antioxidant activity measured by DPPH, ABTS, and FRAP assays, according to the results of the cluster analysis, were included in one separate cluster together with the total content of phenolic compounds, and also significantly depended on the host tree species. It is known that the antioxidant potential of plants is largely determined by the qualitative and quantitative composition of phenolic compounds [40]. However, as noted above, some organic acids present in mistletoe (citric acid and succinic acid) also have antioxidant activity [36]. In addition, some studies have shown that ursolic and oleanolic acids also show antioxidant effects [41]. However, since 70% water–ethanol extracts were used for the analysis of antioxidant activity, extracts of the same mistletoe samples were characterized by high antioxidant activity, in which a high content of phenolic compounds (flavonoids and phenolic acids) was determined. These were mistletoe samples from *B. pendula*, *A. platanoides*, *C. monogyna*, and *S. aucuparia*.

### 3.2. Effect of Mistletoe Organ Type on the Content of Phytochemicals

The results of this study showed a significant influence of the mistletoe organ type on the content of the studied phytocomponents and antioxidant activity (Table 1, Table 2, Table 3, Table 4 and Table 5). Mistletoe fruits were distinguished by an approximately three times higher total content of phenolic compounds and 1.5–3 times higher antioxidant activity compared to leaves and stems. The average total content of phenolic compounds in fruits was about 22 mg GAE g^–1^ DW. Previous studies present contradictory data on the ratio of the total content of phenolic compounds in leaves, stems, and fruits of mistletoe. For example, in [32], it was shown that the total content of phenolic compounds in mistletoe fruits was two times lower compared to leaves. However, Majeed et al. (2021) found that the distribution of phenolic compounds in the organs of mistletoe strongly depended on the type of host tree. For example, for ethanol extracts of mistletoe fruits from *Poplus ciliata*, the total content of phenolic compounds was two times higher compared to leaves [30]. In the present study, it was also found that the relationship of two factors (the type of host tree and mistletoe organ) had a significant effect on the content of phenolic compounds. It is worth noting that the total content of flavonoids, hydroxycinnamic acids, as well as the content of individual phenolic acids and flavonoids (with the exception of kaempferol derivative) were significantly higher in leaves and stems compared to fruits. In the work cited above [30], for the same extracts of mistletoe fruits from *Populus ciliata*, the total content of flavonoids in the leaves was higher compared to fruits. It is believed that, in plants, flavonoid biosynthesis is carried out in all tissues and plays an important role in the interaction of the plant with the environment and/or other organisms [42]. However, the distribution of flavonoids between organs can be influenced not only by the activity of their biosynthesis in a particular organ, but also by the fact that flavonoids can also be transported from where they are synthesised to other parts of the plant [43].

Saponins are usually distributed in plants in tissue-specific and development-dependent manners, which may be helpful to defend against pests and pathogens [44]. In the present study, the type of mistletoe organ significantly influenced the content of triterpenic acids, which was higher in leaves and stems of mistletoe compared to fruits (Table 3). The results obtained in this study are consistent with the data presented in [45], in which it was also shown that the content of oleanolic and betulinic acids was higher in leaves compared to mistletoe fruits. Similar to flavonoids, it has been shown that the content of triterpenic acids in a plant organ depends on several processes, in particular, on direct biosynthesis and transport from other organs [46].

A high content of organic acids was found in both leaves and fruits of mistletoe. As noted above, there is no sufficient information in previous studies on the content of organic acids in mistletoe and no data on their distribution between different organs. It is known that the composition of accumulated organic acids varies depending on the organ type, age of the plant, and type of tissue. The high accumulation of organic acids in photosynthetic plant tissues is most likely due to their important role as intermediate products of photosynthesis [47]. The various organic acids present in fruits are involved in numerous and often unrelated metabolic processes and include compounds that act as intermediates in various metabolic pathways. For example, organic acids act as precursors for the synthesis of amino acids, many plant hormones (for example, auxins, gibberellins, and salicylic acid), fatty acids, a large number of secondary metabolites, and some components of the cell wall [48].

### 3.3. Mistletoe as a Resource of Biologically Active Compounds

For a long time, mistletoe was considered primarily a source of polypeptides, such as viscotoxins and lectins. Recent studies have shown that leaves, stems, and fruits of mistletoe also contain other important phytocomponents (phenolic compounds, terpenoids, alkaloids, amino acids, and organic acids) with high pharmacological or nutritional value [11,12,15,20,21,26,49]. According to the results of the present research, it can be noted that mistletoe growing on *B. pendula*, *A. platanoides*, *C. monogyna*, and *S. aucuparia* is promising as a potential source of phenolic compounds. In samples from these tree species, a high content was noted of both some groups of phenolic compounds and individual phenolic acids and flavonoids. The leaves and stems of mistletoe from *T. cordata* were distinguished by a high content of triterpenic acids (oleanolic, ursolic, and betulinic). The results of the qualitative and quantitative composition of organic acids allow mistletoe to be considered a raw material for the production of food and feed additives. Mistletoe fruits can be used to produce extracts with high antioxidant activity. Especially promising from an economic and ecological point of view is the use of mistletoe bushes as plant raw materials, which remain after the sanitary pruning of urban and garden trees. However, for the use of mistletoe growing in urban conditions, additional studies are required to assess its safety, in particular, the content of inorganic and organic pollutants in its tissues.

## 4. Materials and Methods

### 4.1. Plant Material

Samples of mistletoe plants (*Viscum album* L.) were collected during their fruiting period from January to February 2022 on the territory of Kaliningrad (54°44′56″ N, 20°30′55″ E). Plant samples were collected from 8 species of host trees, namely, *Tilia cordata* Mill., *Acer platanoides* L., *Acer saccharinum* L., *Populus nigra* L., *Salix alba* L., *Crataegus monogyna* Jacq., *Sorbus aucuparia* L., and *Betula pendula* Roth. These species, according to previous studies [3], are the most affected by mistletoe in the city. For research, four trees of each species were selected, characterized by an average degree of mistletoe damage (from 10 to 30 bushes). Several mistletoe samples (from 4 to 6), collected from each tree, were combined into one averaged sample. Samples collected from different trees of the same species were analysed separately (*n* = 4). All the plant samples were identified by Dr. A. Pungin. Voucher specimens were deposited in the herbarium of Immanuel Kant Baltic Federal University (KLGU Herbarium).

For analysis, the collected plant material was divided into organs (leaves, stems, and fruits), lyophilized, and crushed to a particle size of less than 0.5 mm.

### 4.2. Determination of Phenolic Compounds

#### 4.2.1. Extract Preparation

The extraction of phenolic compounds was carried out from crushed lyophilized plant material with 70% ethanol. A sample of plant material weighing 1 g was placed in a round-bottomed flask with the addition of about 40 mL of 70% ethanol and heated at 60 °C in a water bath under reflux for 1 h. The mixture was then filtered into a measuring flask. The extraction procedure was repeated three times. The resulting portions of the extract were combined and brought to 100 mL with 70% ethanol.

#### 4.2.2. Determination of Total Contents of Some Groups of Phenolic Compounds

The total content of phenolic compounds was determined by spectrophotometric method using the Folin–Ciocalteu reagent [50]. The reaction was carried out in a flat-bottom 96-well microplate. Twenty microliters of the extract or standard and 100 µL of Folin–Ciocalteu reagent were added to each well. The mixture was kept for 4 min, and then 75 µL of Na_2_CO_3_ (7.5%, *w*/*w*) was added. After incubation for 2 h in the dark at room temperature, optical absorption was recorded at 765 nm using a microplate reader (CLARIOstar, BMG Labtech, Germany). Gallic acid was used as a standard. The total content of phenolic compounds was determined according to a calibration curve and expressed in mg of gallic acid equivalents per gram of dry weight (mg GAE g^–1^ DW).

The total content of flavonoids was determined by complexation reaction with aluminium chloride in the presence of sodium acetate according to [50], with some modifications. The reaction mixture consisted of 20 µL of the extract or standard, 10 µL of a 10% aluminium chloride solution, 10 µL of 1 M sodium acetate, and 130 µL of 96% ethanol. The mixture was incubated for 40 min in the dark at room temperature. Optical absorption was recorded at 415 nm using a microplate reader (CLARIOstar, BMG Labtech, Germany). Quercetin was used as a standard. The total content of flavonoids was expressed in mg of quercetin equivalents per gram of dry weight (mg QE g^–1^ DW).

The total content of hydroxycinnamic acids was determined by the reaction with Arno’s reagent according to [51], with some modifications. The reaction was carried out in a flat-bottom 96-well microplate. Twenty microliters of the extract or standard, 40 µL of 0.5 M hydrochloric acid, 40 µL of Arno’s reagent (a mixture of 10% NaNO_2_ and 10% NaMoO_4_ at the ratio 1:1), 40 µL of 8.5% NaOH, and 60 µL of H_2_O were added to each well. Optical absorption was recorded at 525 nm using a microplate reader (CLARIOstar, BMG Labtech, Germany). Chlorogenic acid was used as a standard. The total content of hydroxycinnamic acids was determined according to a calibration curve and expressed in mg equivalents of chlorogenic acid per gram of dry weight (mg CAE g^–1^ DW).

#### 4.2.3. High-Performance Liquid Chromatography with Diode-Array Detection (HPLC-DAD) Analysis of Individual Phenolic Compounds

Before HPLC analysis, the extracts prepared as described above were filtered and concentrated on a rotary evaporator. The resulting extract was centrifuged at 4500 g for 15 min, and the supernatant was filtered through a syringe filter (0.22 µm). The separation of substances was carried out on a Shimadzu LC-20 Prominence chromatograph with a Shimadzu SPD20MA diode matrix detector and a Phenomenex Luna column (C18 250 × 4.6 mm, 5 µm). As components of the mobile phase, a mixture of solvents was used, water/acetic acid 99.5/0.5 (solvent A) and acetonitrile (B). Gradient mode was used during separation: 0 min—95% A, 5% B; 3 min—88% A, 12% B; 46 min—75% A, 25% B; 49.5 min—10% A, 90% B; 52 min—10% A, 90% B; 52.7 min—95% A, 5% B; 59 min—95% A, 5% B. The flow rate was 0.85 mL/min, the column temperature was 40 °C; the sample volume was 20 µL. Detection was carried out in the wavelength range of 180–900 nm. The identification of compounds was carried out by comparing the retention time of peaks and UV spectra obtained on chromatograms with the corresponding parameters of chromatographically pure sample standards. Chromatograms were processed in the LabSolutions program. Quantitative analysis of the studied flavonoids was carried out using calibration graphs plotted in the concentration range of 10–100 µg mL^–1^. The following standards were used in the study: caftaric acid, chicoric acid, chlorogenic acid, p-coumaric acid, rosmarinic acid, sinapic acid, trans-caffeic acid, 3,4-dihydroxybenzoic acid, gallic acid, ellagic acid, luteolin 7-O-glucoside, apigenin 7-O-glucoside, apigenin 7-O-glucuronide, quercetin 3-O-rutinoside, quercetin 3-β-D-glucoside, kaempferol 3-O-glucoside, isorhamnetin, baicalin, diosmin, and catechin. All standards were purchased from Sigma-Aldrich (Sigma-Aldrich Rus, Moscow, Russia).

### 4.3. Determination of Triterpenic Acids

#### 4.3.1. Extract Preparation

The extraction of triterpenic acids was carried out from crushed lyophilized plant material with acetone. A sample of plant material weighing 1 g was placed in a round-bottomed flask with 50 mL of acetone and heated at 60 °C in a water bath under reflux for 1 h. The mixture was then filtered into a measuring flask. The extraction procedure was repeated three times. The obtained portions of the extract were combined and then concentrated on a rotary evaporator and the volume of the extract was brought to 2 mL.

#### 4.3.2. High-Performance Thin Layer Chromatographic Analysis of Triterpenic Acids

The plant samples were analysed using high-performance thin-layer chromatography (HPTLC) by applying 0.5 µL of each sample to a 10 × 10 cm HPTLC plate with pre-applied silica gel 60 F254 (Merck, Darmstadt, Germany) according to [52], with some modifications. Preliminary derivatization of the HPTLC plate was carried out using a 1% solution of iodine in chloroform (*w*/*v*), which was then kept in the dark for 15 min. After drying, the plates were developed in the CAMAG twin-trough glass chamber at room temperature (25 ± 2 °C) and humidity (65 ± 5%) using a mobile phase comprising hexane–ethyl acetate–acetone–toluene in a ratio of 8.2:1.8:0.2:0.2 (*v*/*v*). The plates were postderivated using a 10% aqueous solution of sulfuric acid for 10 s, and then the dried plates were heated at 110 °C for 5 min. After the strips were developed, the plates were immediately processed at 365 nm using the Sorbfil TLC Videodensitometer^®^ (Sorbfil, Russia). To assess the separation of triterpenic acids, a mixture of oleanolic, ursolic, and betulinic acids was applied to each plate. To calibrate and evaluate the range of linearity, the initial solution of each acid (1 mg mL^–1^) was applied to the plate for HPTLC in an amount of 0.5, 1.0, 1.5, 2.0, 2.5, and 3.0 µL to obtain a linearity range of 0.5–3 µg/point. The concentration of triterpenic acids was calculated using calibration curves with the regression equation and the corresponding peak area. The content of triterpenic acids was expressed in mg per gram of dry weight (mg g^–1^ DW).

### 4.4. Determination of Organic Acids

#### 4.4.1. Extract Preparation

For the extraction of organic acids, 30–40 mL of bidistilled water heated to 70 °C was added to the plant material and kept for 20 min. After that, the homogenates were transferred to measuring flasks and the volume was brought to 50 mL with bidistilled water. The resulting extract was filtered first through a paper filter, and then a 0.45 µm cellulose acetate filter.

#### 4.4.2. Capillary Electrophoretic Analysis of Organic Acids

The content of organic acids in plant samples was determined by capillary electrophoresis on Kapel-105/105M (Lumex, St. Petersburg, Russia) with high negative polarity [53]. Before the analyses, the capillary was conditioned for 30 min with 0.1 M NaOH and 10 min with water. Additionally, the capillary was washed for 3 min with distilled water, 5 min with 0.1 M NaOH, 5 min with water, and 5 min with buffer solution before each start. A solution consisting of a phosphate buffer solution (concentration of phosphate ions 95 mmol L^–1^) and cetriltrimethylammonium bromide (0.1 mmol L^–1^) was used as a buffer. Applied voltage was −20 kV. Detection was carried out indirectly at 190 nm. Quantitative determination was carried out using calibration curves. Solutions of fixed concentrations of oxalic, formic, fumaric, succinic, citric, propionic, malic, and sorbic acids were used as standards.

### 4.5. Determination of Antioxidant Activity

To determine the antioxidant activity, the extracts prepared as described in Section 4.2.1 were used. The antioxidant activity of the extracts was determined by the ability to capture radicals of 2,2-diphenyl-1-picrylhydrazyl (DPPH) and 2,2’-azino-bis(3-ethylbenzthiazolino-6-sulfonic acid (ABTS), as well as by the reducing power when interacting with the Fe(III)-2,4,6-tripyridyl-s-triazine complex (FRAP), according to [54].

When determining the antioxidant activity by the DPPH method, 20 µL of extract or standard solution was mixed with 300 µL of a 0.1 mM solution of 2,2-diphenyl-1-picrylhydrazyl. The mixture was incubated in the dark at room temperature for 30 min. A decrease in optical absorption compared to the control was recorded at 515 nm.

When determining the antioxidant activity by the ABTS method, a solution of ABTS radical was pre-prepared. ABTS radical was generated by mixing aliquots of a 7.0 mM ABTS solution and 2.45 mM potassium persulfate solution. The solution was kept for 16 h in a dark place at room temperature. To carry out the reaction, 20 µL of extract or standard was added to 300 µL of the prepared ABTS radical cation solution. The optical absorption was measured at 734 nm after incubation of the mixture for 15 min at 37 °C in the dark.

To determine the regenerating power of the extracts, a freshly prepared FRAP reagent was used, prepared by mixing 10 parts of 0.3 M acetate buffer (pH 3.6), one part of a 10 mM solution of 2,4,6-tripyridyl-s-triazine in 40 mM HCl, and one part of an aqueous 20 mM solution of ferric chloride FeCl_3_×6H_2_O. The reaction was started by mixing 300 mL of FRAP reagent and 20 mL of the studied extract or standard solution. The reaction time was 10 min at 37 °C in the dark. An increase in optical absorption compared to the control was recorded at 593 nm.

When measuring antioxidant activity using the DPPH, ABTS, and FRAP methods, solutions of Trolox (6-hydroxy-2,5,7,8-tetramethylchroman-2-carboxylic acid) of known concentration were used as a standard solution. The results of the analyses are expressed in mg of Trolox equivalents (mg TE g^–1^). Spectrophotometric measurements were carried out using a CLARIOstar microplate reader (BMG Labtech, Germany).

### 4.6. Statistical Analysis

The statistical processing of experimental data was carried out using OriginPro 2019b (OriginLab Corporation, Northampton, MA, USA). To assess the influence of factors (host tree species and organ of mistletoe), the effect of their interaction, and the significance of the differences between the means, two-way ANOVA was performed, followed by the use of the Tukey test, with significance set at *p* ≤ 0.05. The results in the tables are reported as the mean ± standard deviation. The heat map and clusters were built based on the normalized mean values (*n* = 4) of the analysed variables. Euclidean distance was used as a measure of similarity.

## Figures and Tables

**Table 1:** Total content of phenolic compounds, flavonoids, and hydroxycinnamic acids in mistletoe, depending on host tree species and type of mistletoe organ.

Factors	Level	TPC, mg GAE g^–1^ DW	TFC, mg QE g^–1^ DW	THA, mg CAE g^–1^ DW
Main Effects ^1^				
Host tree species (S)	*T. cordata*	11.43 ± 2.01 ab	0.83 ± 0.16 bcd	1.43 ± 0.23 bcd
	*A. platanoides*	13.17 ± 2.69 a	0.97 ± 0.34 abc	1.64 ± 0.26 bc
	*A. saccharinum*	9.74 ± 1.63 b	0.50 ± 0.10 d	0.99 ± 0.19 d
	*P. nigra*	10.43 ± 2.15 ab	0.57 ± 0.10 cd	0.89 ± 0.11 d
	*S. alba*	12.57 ± 2.88 a	0.77 ± 0.18 cd	1.10 ± 0.20 cd
	*C. monogyna*	13.44 ± 1.99 a	1.24 ± 0.46 a	1.83 ± 0.59 b
	*S. aucuparia*	12.89 ± 2.69 a	1.20 ± 0.33 ab	1.24 ± 0.33 cd
	*B. pendula*	12.34 ± 1.09 a	1.26 ± 0.46 a	2.48 ± 0.64 a
Organ of mistletoe (O)	Stems	7.16 ± 1.67 b	0.78 ± 0.22 b	1.58 ± 0.37 a
	Leaves	6.89 ± 1.48 b	1.21 ± 0.55 a	1.62 ± 0.42 a
	Fruits	21.96 ± 3.59 a	0.75 ± 0.17 b	1.14 ± 0.21 b
Significance	S	<0.001 *	<0.001 *	<0.001 *
	O	<0.001 *	<0.001 *	<0.001 *
	S * O	<0.001 *	<0.001 *	<0.001 *

^1^ Data were evaluated via two-way ANOVA, with factors host tree species and organ of mistletoe, followed by a Tukey HSD test. Identical letters indicate that values do not differ significantly. Asterisks (*) indicate significantly influential factors. TPC—total phenolics content; TFC—total flavonoid content; THA—total hydroxycinnamic acid content; GAE—gallic acid equivalents; QE—quercetin equivalents; CAE—chlorogenic acid equivalents.

**Table 2:** Content of individual phenolic acids and flavonoids in mistletoe, depending on host tree species and type of mistletoe organ.

Factors	Level	Content of Individual Phenolic Compounds, mg g^–1^ DW					
		NeoChl	Chl	Caff	IsoRham	Quer-D	Kaem-D
Main Effects ^1^							
Host tree species (S)	*T. cordata*	0.23 ± 0.08 bc	0.30 ± 0.07 bc	0.032 ± 0.006 b	0.056 ± 0.006 bcd	0.10 ± 0.03 a	0.085 ± 0.007 cde
	*A. platanoides*	0.37 ± 0.05 ab	0.43 ± 0.06 a	0.046 ± 0.011 a	0.059 ± 0.013 bcd	0.090 ± 0.007 a	0.089 ± 0.025 bcd
	*A. saccharinum*	0.10 ± 0.02 cd	0.18 ± 0.03 d	0.032 ± 0.012 b	0.030 ± 0.004 e	0.047 ± 0.006 c	0.054 ± 0.007 e
	*P. nigra*	0.09 ± 0.02 d	0.11 ± 0.02 d	0.021 ± 0.003 c	0.044 ± 0.008 cde	0.061 ± 0.016 bc	0.066 ± 0.015 de
	*S. alba*	0.11 ± 0.03 cd	0.13 ± 0.03 d	0.025 ± 0.009 bc	0.042 ± 0.004 de	0.084 ± 0.027 ab	0.081 ± 0.037 cde
	*C. monogyna*	0.34 ± 0.05 ab	0.38 ± 0.08 ab	0.035 ± 0.006 b	0.074 ± 0.024 ab	0.089 ± 0.028 ab	0.12 ± 0.02 b
	*S. aucuparia*	0.19 ± 0.03 cd	0.18 ± 0.02 cd	0.020 ± 0.004 c	0.063 ± 0.010 bc	0.083 ± 0.018 ab	0.11 ± 0.02 bc
	*B. pendula*	0.41 ± 0.06 a	0.38 ± 0.06 ab	0.032 ± 0.009 b	0.088 ± 0.031 a	0.090 ± 0.034 a	0.17 ± 0.04 a
Organ of mistletoe (O)	Stems	0.28 ± 0.03 a	0.29 ± 0.03 a	0.033 ± 0.011 a	0.059 ± 0.023 ab	0.084 ± 0.025 a	0.094 ± 0.05 a
	Leaves	0.27 ± 0.03 a	0.30 ± 0.04 a	0.031 ± 0.012 ab	0.065 ± 0.029 a	0.096 ± 0.031 a	0.11 ± 0.05 a
	Fruits	0.14 ± 0.01 b	0.17 ± 0.01 b	0.026 ± 0.009 b	0.047 ± 0.008 b	0.063 ± 0.016 b	0.090 ± 0.02 a
Significance	S	<0.001 *	<0.001 *	<0.001 *	<0.001 *	<0.001 *	<0.001 *
	O	<0.001 *	<0.001 *	<0.001 *	<0.001 *	<0.001 *	<0.001 *
	S * O	<0.001 *	<0.001 *	<0.001 *	<0.001 *	<0.001 *	<0.001 *

^1^ Data were evaluated via two-way ANOVA, with factors host tree species and organ of mistletoe, followed by a Tukey HSD test. Identical letters indicate that values do not differ significantly. Asterisks (*) indicate significantly influential factors. NaoChl—neochlorogenic acid; Chl—chlorogenic acid; Caff—caffeic acid; IsoRham—isorhamnetin; Quer-D—quercetin derivative; Kaem-D—kaempferol derivative.

**Table 3:** Content of triterpenic acids in mistletoe, depending on host tree species and type of mistletoe organ.

Factors	Level	Content of Triterpenic Acids, mg g^–1^ DW		
		Ursolic	Oleanolic	Betulinic
Main Effects ^1^				
Host tree species (S)	*T. cordata*	1.09 ± 0.27 a	6.18 ± 0.89 a	0.91 ± 0.39 ab
	*A. platanoides*	0.97 ± 0.37 ab	4.39 ± 1.32 bc	0.54 ± 0.18 bc
	*A. saccharinum*	1.03 ± 0.33 ab	4.50 ± 1.28 bc	0.63 ± 0.24 bc
	*P. nigra*	0.84 ± 0.23 ab	4.96 ± 1.07 b	1.04 ± 0.39 a
	*S. alba*	1.12 ± 0.53 a	4.58 ± 1.17 bc	0.92 ± 0.26 ab
	*C. monogyna*	0.64 ± 0.15 b	2.35 ± 0.18 d	0.51 ± 0.24 bc
	*S. aucuparia*	0.80 ± 0.25 ab	3.18 ± 1.12 cd	0.27 ± 0.07 c
	*B. pendula*	0.84 ± 0.35 ab	2.97 ± 0.96 cd	0.52 ± 0.21 bc
Organ of mistletoe (O)	Stems	1.14 ± 0.33 a	4.77 ± 1.53 a	0.97 ± 0.35 a
	Leaves	1.09 ± 0.15 a	5.02 ± 1.47 a	0.80 ± 0.27 b
	Fruits	0.53 ± 0.11 b	2.62 ± 0.89 b	0.24 ± 0.08 c
Significance	S	<0.001 *	<0.001 *	<0.001 *
	O	<0.001 *	<0.001 *	<0.001 *
	S * O	<0.001 *	<0.001 *	<0.001 *

^1^ Data were evaluated via two-way ANOVA, with factors host tree species and organ of mistletoe, followed by a Tukey HSD test. Identical letters indicate that values do not differ significantly. Asterisks (*) indicate significantly influential factors.

**Table 4:** Content of organic acids in mistletoe depending on host tree species and type of mistletoe organ.

Factors	Level	Content of Organic Acids, mg g^–1^ DW							
		Succinic	Citric	Oxalic	Formic	Fumaric	Propionic	Malic	Sorbic
Main Effects ^1^									
Host tree species (S)	*T. cordata*	3.09 ± 0.47 c	1.27 ± 0.26 c	0.29 ± 0.04 bc	1.11 ± 0.27 b	0.019 ± 0.001 bc	0.23 ± 0.09 c	-	0.047 ± 0.011 c
	*A. platanoides*	4.02 ± 0.52 bc	1.71 ± 0.66 c	0.26 ± 0.03 bc	0.19 ± 0.01 c	0.007 ± 0.001 c	-	1.66 ± 0.33 a	0.63 ± 0.08 a
	*A. saccharinum*	3.32 ± 1.04 c	2.97 ± 0.43 ab	0.38 ± 0.10 b	0.26 ± 0.06 c	0.021 ± 0.004 b	0.37 ± 0.07 bc	-	0.30 ± 0.04 b
	*P. nigra*	8.07 ± 0.68 a	3.75 ± 0.36 a	0.23 ± 0.07 c	0.22 ± 0.01 c	0.036 ± 0.009 a	0.81 ± 0.19 b	1.49 ± 0.26 a	0.27 ± 0.05 b
	*S. alba*	8.39 ± 0.47 a	2.16 ± 0.46 bc	0.90 ± 0.10 a	3.10 ± 0.49 a	0.031 ± 0.010 ab	0.15 ± 0.04 c	-	-
	*C. monogyna*	5.35 ± 0.95 bc	1.65 ± 0.18 c	0.92 ± 0.06 a	0.44 ± 0.07 bc	0.019 ± 0.004 bc	3.66 ± 1.47 a	1.75 ± 0.37 a	0.061 ± 0.006 c
	*S. aucuparia*	5.99 ± 1.20 ab	1.94 ± 0.10 c	0.90 ± 0.10 a	0.83 ± 0.27 b	0.016 ± 0.005 bc	-	-	0.16 ± 0.02 bc
	*B. pendula*	4.34 ± 1.21 bc	1.53 ± 0.16 c	0.94 ± 0.07 a	1.33 ± 0.62 b	0.017 ± 0.003 bc	0.48 ± 0.11 bc	-	-
Organ of mistletoe (O)	Stems	4.16 ± 0.42 b	1.51 ± 0.12 b	0.60 ± 0.09 a	0.89 ± 0.22 b	0.017 ± 0.006 b	0.54 ± 0.18 b	0.35 ± 0.09 b	0.20 ± 0.04 a
	Leaves	5.94 ± 0.49 a	2.52 ± 0.27 a	0.57 ± 0.05 a	1.62 ± 0.59 a	0.031 ± 0.012 a	1.13 ± 0.37 a	1.39 ± 0.34 a	0.075 ± 0.009 b
	Fruits	5.86 ± 0.46 a	2.33 ± 0.51 a	0.64 ± 0.06 a	0.30 ± 0.06 c	0.015 ± 0.007 b	1.18 ± 0.21 a	-	0.27 ± 0.09 a
Significance	S	<0.001 *	<0.001 *	<0.001 *	<0.001 *	<0.001 *	<0.001 *	<0.001 *	<0.001 *
	O	<0.001 *	<0.001 *	<0.001 *	<0.001 *	<0.001 *	<0.001 *	<0.001 *	<0.001 *
	S * O	<0.001 *	<0.001 *	<0.001 *	<0.001 *	<0.001 *	<0.001 *	<0.001 *	<0.001 *

^1^ Data were evaluated via two-way ANOVA, with factors host tree species and organ of mistletoe, followed by a Tukey HSD test. Identical letters indicate that values do not differ significantly. Asterisks (*) indicate significantly influential factors.

**Table 5:** Antioxidant activity of extracts from the leaves, stems, and fruits of mistletoe harvested from different host tree species.

Factors	Level	Antioxidant Activity, mg TE g^–1^		
		DPPH	ABTS	FRAP
Main Effects ^1^				
Host tree species (S)	*T. cordata*	5.63 ± 1.11 a	18.72 ± 3.07 bc	7.82 ± 1.14 ab
	*A. platanoides*	5.74 ± 1.02 a	19.86 ± 2.76 abc	7.67 ± 1.28 ab
	*A. saccharinum*	2.58 ± 0.33 b	16.80 ± 2.32 bc	4.79 ± 0.61 b
	*P. nigra*	4.14 ± 0.99 ab	14.28 ± 3.06 c	5.74 ± 1.29 b
	*S. alba*	4.90 ± 1.41 ab	19.5 ± 3.89 abc	7.89 ± 1.67 ab
	*C. monogyna*	6.23 ± 0.91 a	21.85 ± 1.86 ab	9.24 ± 0.85 a
	*S. aucuparia*	6.67 ± 1.88 a	20.50 ± 4.61 abc	10.11 ± 2.36 a
	*B. pendula*	4.66 ± 0.17 ab	25.26 ± 2.34 a	9.52 ± 0.66 a
Organ of mistletoe (O)	Stems	2.82 ± 0.17 b	17.30 ± 2.13 b	6.08 ± 0.51 b
	Leaves	2.77 ± 0.20 b	16.31 ± 4.36 b	4.34 ± 0.27 b
	Fruits	9.62 ± 1.61 a	25.16 ± 3.07 a	13.12 ± 0.69 a
Significance	S	<0.001 *	<0.001 *	<0.001 *
	O	<0.001 *	<0.001 *	<0.001 *
	S * O	<0.001 *	<0.001 *	<0.001 *

^1^ Data were evaluated via two-way ANOVA, with factors host tree species and organ of mistletoe, followed by a Tukey HSD test. Identical letters indicate that values do not differ significantly. Asterisks (*) indicate significantly influential factors. DPPH—antioxidant activity determined by 2,2-diphenyl-1-picrylhydrazyl assay; ABTS—antioxidant activity determined by 2,2′-azino-bis(3-ethylbenzothiazoline-6-sulfonic acid) assay; FRAP—ferric reducing/antioxidant power; TE—Trolox equivalent.

## Data Availability

Not applicable.
